# The Complete Mitochondrial Genome of *Portunion sinensis* (Crustacea: Isopoda) and Its Phylogenies

**DOI:** 10.3390/biology15030282

**Published:** 2026-02-04

**Authors:** Teng Huang, Xiaowan Ma, Shengping Zhong, Jie Chen, Dewei Cheng, Xuyang Chen, Dong Yang, Lixing Huang, Theerakamol Pengsakul, Ying Qiao, Wenhong Li

**Affiliations:** 1 College of Animal Science and Technology, Guangxi University, Nanning 530200, China; 19927530646@163.com (T.H.); maxiaowan@4io.org.cn (X.M.); chenxuyang@4io.org.cn (X.C.); yangdong201602@163.com (D.Y.); 2 Key Laboratory of Tropical Marine Ecosystem and Bioresource, Fourth Institute of Oceanography, Ministry of Natural Resources, Beihai 536000, China; chenjie@4io.org.cn (J.C.); chengdewei@4io.org.cn (D.C.); 3 Institute of Marine Drugs, Guangxi University of Chinese Medicine, Nanning 530200, China; 4 Key Laboratory of Healthy Mariculture for the East China Sea, Ministry of Agriculture, Fisheries College, Jimei University, Xiamen 361000, China; lixinghuang@outlook.com; 5 Health and Environmental Research Center, Faculty of Environmental Management, Prince of Songkla University, Hat Yai 90110, Songkhla, Thailand; theerakamol.p@psu.ac.th

**Keywords:** *Portunion*, *Scylla paramamosain*, mitochondrial genome, parasite, phylogeny

## Abstract

In this study, we conducted molecular identification and complete mitochondrial genome annotation of isopod parasites isolated from mud crabs (*Scylla paramamosain*) in the China Sea. The specimen was identified as *Portunion sinensis*, a recently described species of the family Entoniscidae. *P. sinensis* is characterized by an anterior thorax positioned vertically and posterior ventral processes directed backwards; the female’s marsupium is formed by the close adhesion of oostegites to the host membrane; the first oostegite is differentiated into three parts; the pleopoda have well-developed lamellar and pleural lamellae; and two ventral ovarian processes are present along with a pair of dorsal processes. The complete mitochondrial genome of the parasite is 14,603 bp in length, containing 35 genes (13 protein-coding genes, 20 transfer RNAs, and 2 ribosomal RNAs) as well as a putative control region (CR) located between the *cob* and *nad1* genes. This study represents the initial characterization of the mitochondrial genome of *P. sinensis*. These findings provide molecular validation of its status as an independent species and lay the groundwork for the future identification of parasites within the genus *Portunion*. These data provide a necessary molecular reference for future research into the systematics and evolutionary history of decapod-infesting parasites.

## Simple Summary

The mud crab (*Scylla paramamosain*) is an economically important crustacean species. This study reports the first complete mitochondrial genome of the parasitic isopod *Portunion sinensis* (family Entoniscidae) infecting this crab. We conducted a detailed analysis of its genomic architecture, revealing a compact genome and unique structural characteristics. As the first representative mitochondrial genome for the family Entoniscidae, this work fills a critical gap in molecular data for the superfamily Bopyroidea. These findings not only provide essential genetic resources for species identification but also serve as a key reference for elucidating the mitochondrial structure and phylogenetic relationships of parasitic crustaceans.

## 1. Introduction

The mud crab, *Scylla paramamosain* (Portunoidea, Brachyura), is an economically important crustacean species widely distributed along the coast of southern China. However, its wild and cultivated populations are frequently infested by parasitic diseases. *Portunion sinensis* is a parasitic isopod that specifically infects *S. paramamosain* and was formally described in recent taxonomic studies [1]. Taxonomically, this parasite belongs to the subfamily Entioninae within the family Entoniscidae (Superfamily Bopyroidea). Members of the superfamily Bopyroidea exhibit extreme sexual dimorphism and a holoparasitic lifestyle that has a profound effect on their host [2]. They predominantly inhabit the gill cavity, abdomen, or hemocoel of crustaceans, leading to diapause, failure of ecdysis, gonadal development inhibition, and parasitic castration [3]. Specifically, *P. sinensis* inhabits the hemocoel of *S. paramamosain*, posing a potential threat to the mud crab aquaculture industry.

Accurate species identification of the genus *Portunion*, and indeed the family Entoniscidae, is highly challenging due to their extreme morphological specialization. To adapt to a reproductive lifestyle within the host, the female *Portunion* sp. has completely lost the typical morphological characteristics of an isopod [4]. The female is enveloped by a sheath formed by the host [5], which connects to the exterior via an exit pore in the host’s gill cavity. In contrast, the male individuals are dwarfed and segmented; they attach to the female and remain morphologically recognizable as typical isopods. The epicaridium larvae leave the host through the gill pores, utilizing copepods as intermediate hosts before eventually metamorphosing into infective larvae [2]. Currently, the World Register of Marine Species (WoRMS) recognizes eight valid *Portunion* species. Researchers typically rely on complex morphological characteristics for classification, such as the host species, the orientation of the female’s thoracic protrusions, the fusion of the male’s cephalothorax, the second antenna, abdominal spines, and the characteristics of the sixth pereopod of the epicaridium larvae [6,7]. However, relying solely on these reduced morphological traits can be problematic, necessitating the urgent use of molecular markers for precise identification and phylogenetic analysis.

Despite the taxonomic diversity of the genus, molecular data for the family Entoniscidae are scarce. While mitochondrial genomes have been widely sequenced for other isopod lineages (e.g., the suborders Cymothoida and Oniscidea), genomic resources for the family Entoniscidae are currently restricted to partial sequences of specific markers (e.g., *cox1* and *18S* rRNA), leaving the complete mitogenomic architecture of this family unknown. Currently, molecular records for the genus *Portunion* are limited to a single *cox1* DNA fragment from *P. conformis*. Even within the broader superfamily Bopyroidea, species with complete mitochondrial gene sequences are rare (e.g., *Gyge ovalis*) [8]. The complete mitochondrial genome serves as a distinct and accessible molecular marker that facilitates not only parasite species identification [9] and phylogenetic analyses [10], but also investigations into population genetic structure [11].

To bridge this research gap, we sequenced and assembled the complete mitochondrial genome of *P. sinensis* and explored its phylogenetic position at the mitogenomic level. This study contributes the first complete mitogenome for the genus *Portunion*, enriching the limited molecular data available for the family Entoniscidae, and providing key evidence for an in-depth understanding of the genomic variation characteristics and phylogenetic relationships of isopod species. These data offer a necessary molecular reference for future research into the taxonomy and evolutionary history of parasitic crustaceans.

## 2. Materials and Methods

### 2.1. Source of Materials

**Figure 1:**
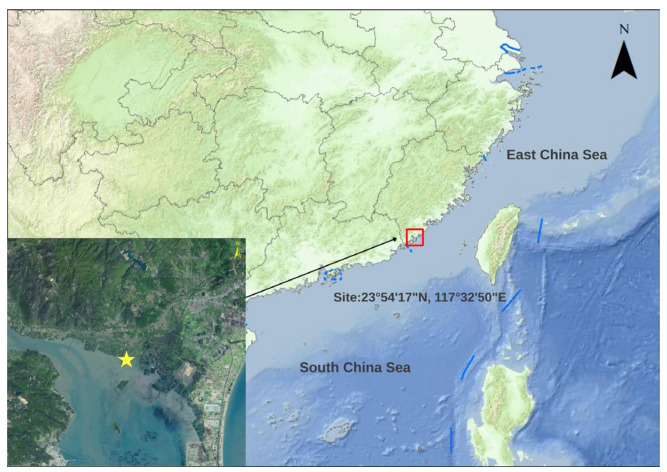
Collecting sites of the *Portunion sinensis* in Zhangpu, Fujian, China. The yellow star indicates the specific collection locality.

**Figure 2:**
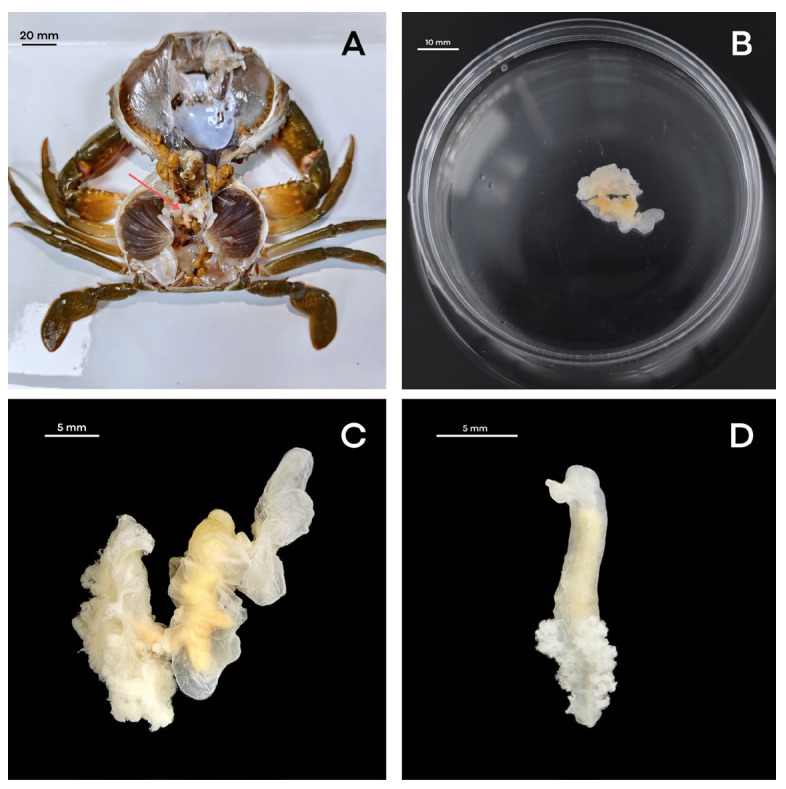
Dissection and observation of morphology of *Portunion sinensis*. (**A**) Dissection of the mud crab; (**B**) Long shot of the female *P. sinensis*. (**C**) V shape of the female *P. sinensis*; (**D**) juvenile of the female *P. sinensis*. Scale bars: (**A**) = 20 mm; (**B**) = 10 mm; (**C**,**D**) = 5 mm.

For this study, we collected parasite-infected crabs from the natural waters of Zhangpu, Fujian, China (23°54′17″ N, 117°32′50″ E) (Figure 1), the type locality of the parasite, and then transported the crabs to the laboratory. To obtain live parasite samples, crabs were dissected while alive. Humane euthanasia was achieved by immersing the crab in a mixture of ice and water at 0 °C before proceeding with the dissection. The carapace was carefully detached to expose the internal organs, and female parasites were searched for within the host’s visceral mass (Figure 2A). The parasite was then separated from the mud crab’s hemocoel using sterile forceps, rinsed briefly with saline solution to remove residual host tissue from its surface, and placed in a clean Petri dish for subsequent observation (Figure 2B).

### 2.2. Morphological Observation

Specimens were preserved using two methods depending on downstream analysis: individuals for morphological identification were fixed in 75% ethanol, while ovigerous females intended for genomic DNA extraction were preserved in 100% ethanol at −20 °C [12]. Mature female parasites, characterized by a marsupium filled with eggs and embryos, appeared as distinct yellow masses easily distinguishable from host organs. In contrast, juvenile parasites with transparent, empty marsupia required meticulous examination to distinguish them from the host’s gonads. Morphological characteristics were observed under a stereoscopic microscope (SteREO Discovery.V12, Zeiss, Oberkochen, Germany) (Figure 2C,D). Body dimensions were measured as follows: abdominal length (AT), defined as the distance from the thorax–abdomen junction to the posterior extremity; and head–thorax length (HT), defined as the distance from the anterior head margin to the thorax–abdomen junction along the dorsal midline.

### 2.3. Whole Mitochondrial Genome Sequencing

To eliminate genomic contamination from the host, eggs or epicaridium larvae were specifically collected from the female marsupium for DNA extraction [12]. Total genomic DNA was extracted using the MolPure^®^ Marine Animals DNA Kit (Yeasen Biotech, Shanghai, China) following the manufacturer’s protocol. The concentration and purity of the extracted DNA were measured using a Nano 500 micro spectrophotometer (Allsheng, Hangzhou, China). The integrity of the genomic DNA was assessed by 1% agarose gel electrophoresis [13]. A total of approximately 1.5 µg of genomic DNA was used for library construction. Before whole-genome sequencing, the species identity was verified by amplifying the cytochrome c oxidase subunit I (*cox1*) gene [14]. PCR primers were designed targeting conserved motifs identified through an alignment of available *cox1* sequences from related isopod species. The PCR reaction (50 μL) contained 0.05 μg of DNA, 0.4 μM of each primer (Forward: *Portunion*-*cox1*-F 5′-ATGCAACGTTGAATATACTCTACTA-3′; Reverse: *Portunion*-*cox1*-R 5′-AGAGCACTCCCACAAACATCA-3′), 25 μL of 2× EasyTaq^®^ PCR SuperMix (TransGen Biotech, Beijing, China), and 22 μL of ddH_2_O. The thermal cycling conditions were as follows: 95 °C for 7 min; 30 cycles of 95 °C for 30 s, 55 °C for 30 s, and 72 °C for 1 min; and a final extension at 72 °C for 10 min. PCR products were purified and sequenced using Sanger sequencing at BGI (Beijing, China) to confirm the taxonomic identity.

### 2.4. Mitochondrial Genome Structure Annotation and Analysis

Following molecular verification, the genomic DNA was subjected to high-throughput sequencing. DNA libraries with an insert size of 350 bp were constructed using the TruSeq Nano™ Kit (Illumina, San Diego, CA, USA) following the manufacturer’s instructions. Sequencing was performed on the Illumina HiSeq platform at BGI (Beijing, China) (2 × 150 bp paired-end reads). Prior to assembly, raw reads were filtered using fastp to remove adapters [15], poly-N sequences, and low-quality reads (Q-value < 20).

The mitochondrial genome assembly was performed using MITObim (v1.9.1) [16] with the previously amplified *cox1* fragment serving as the reference seed for the iterative mapping process (default parameters, maximum iterations = 100). The resulting contigs were screened using BLASTn (v2.9.0) against the NCBI nucleotide database to identify the complete mitochondrial genome. The assembled circular mitogenome was then annotated using the MITOS2 web server and manually corrected for start/stop codons [17]. Transfer RNA (tRNA) genes were independently validated using tRNAscan-SE (v2.0) [18]. The circular genome map was generated using the Proksee online platform [19].

Nucleotide composition bias was assessed by calculating skew values using the following formulas [20]: AT-skew = (A − T)/(A + T) and GC-skew = (G − C)/(G + C). The GC-skew value measures the relative abundance of guanine (G) versus cytosine (C) on the coding strand, serving as an indicator of strand asymmetry resulting from replication and transcription pressures. To evaluate codon usage bias, Relative Synonymous Codon Usage (RSCU) was calculated according to the formula [21] *RSCU_ij_* = (*X_ij_* × *n_i_*)/*ΣX_ij_*, where *X_ij_* is the frequency of the *j*-th codon for the *i*-th amino acid, and n*_i_* represents the number of synonymous codons (degeneracy) for that amino acid. These analyses were performed using a custom Python 3 script, and the results were visualized using the ggplot2 package in Rstudio [22].

### 2.5. Phylogenetic Analysis

Phylogenetic analysis was performed using the newly sequenced mitogenome of *P. sinensis* and representative species selected from major isopod suborders, with a specific focus on available sequences within the suborder Epicaridea to ensure taxonomic coverage. Specifically, the dataset incorporated all currently available sequences from the genus *Portunion*, comprising a total of 14 complete mitochondrial genomes and 3 partial sequences, with *Ligia oceanica* serving as the outgroup (Table 1).

Gene extraction and matrix compilation were performed using the integrated platform PhyloSuite v1.2.3 [23]. Thirteen protein-coding genes (PCGs) and two ribosomal RNA genes (rRNAs) were retrieved from each dataset. PCG nucleotide sequences were aligned in batches using the “Codon” mode of MAFFT [24] implemented in PhyloSuite. Ambiguously aligned regions and excessive gaps were removed using Gblocks [25]. Ribosomal RNA sequences were aligned with the Q-INS-i algorithm, which incorporates secondary-structure information, and were subsequently trimmed using trimAl [26]. All individual gene alignments were concatenated in PhyloSuite to generate the final supermatrix.

Model selection and partitioning schemes were determined using PartitionFinder 2 [27] under the corrected Akaike Information Criterion (AICc). Maximum Likelihood (ML) phylogenetic analyses were conducted in IQ-TREE [28], with node support evaluated using 1000 ultrafast bootstrap replicates. The resulting phylogenetic trees were visualized and annotated using the ChiPlot online platform (https://www.chiplot.online/ (accessed on 25 January 2026)) [29].

To assess genetic differentiation, aligned *cox1* sequences were imported into MEGA X [30]. Average intra-specific and inter-specific genetic distances were calculated under the Kimura 2-parameter (K2P) model [31], and standard errors (SEs) were estimated using 1000 bootstrap replicates.

## 3. Results

### 3.1. Observation of Morphology and Parasitism

Specimens were identified as *Portunion sinensis* Huang et al., 2025 [1] based on morphological vouchers (75% ethanol) from the same collection batch as the genomic samples. The females exhibited diagnostic characters consistent with the species description: the anterior thorax is vertical, while the posterior ventral processes are directed backwards. The marsupium is formed by the close adhesion of oostegites to the host membrane. The pleopods possess well-developed lamellar and pleural lamellae. Two ventral ovarian processes and a pair of dorsal processes are present (Figure 2C) [1]. Males were not observed in the examined specimens. A voucher specimen was deposited at the Fourth institute of Oceanography, Ministry of Natural Resources, China (Accession No.: Ps-ZP05). Genomic samples were morphologically verified as conspecific with vouchers prior to DNA extraction.

### 3.2. Mitochondrial Genome Analyses

**Figure 3:**
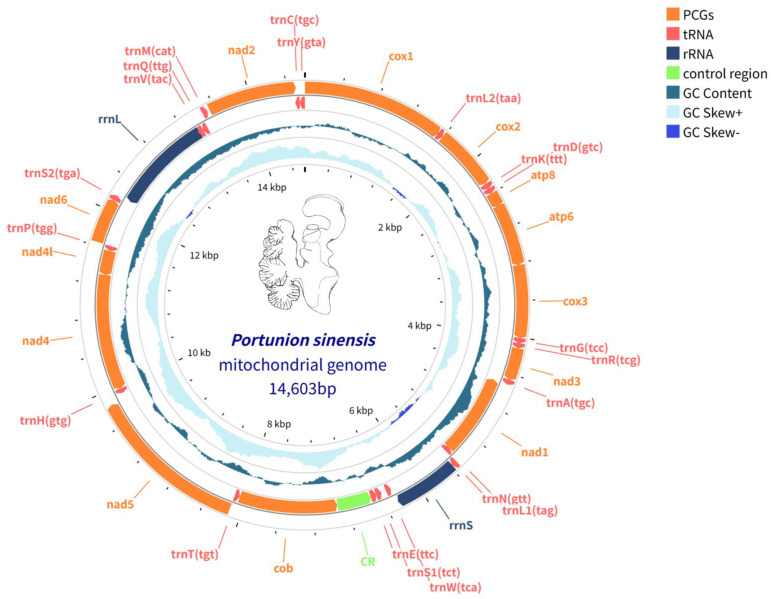
Gene map of the complete mitochondrial genome of *Portunion sinensis*. The complete mitochondrial genome of *Portunion sinensis* contains a total of 35 genes including 13 protein-coding genes, 2 ribosomal RNA genes and 20 transfer RNA genes.

The genome of the parasite was 14,603 bp in length. As illustrated in the genome circle map (Figure 3), the structure of the genome comprises 2 rRNA genes, 13 protein-coding genes, 20 tRNA genes, and a putative control region (CR). The overall base composition is 26.7% A, 31.2% T, 17.8% C and 24.2% G, resulting in a positive GC skew value of 0.15 and an AT skew value of −0.07 (Table 2).

The protein-coding genes of the *P. sinensis* mitochondrial genome have a total length of 10,971 bp, accounting for 75.13% of the total genome, with an AT skew of −0.197 and a GC skew of 0.084. Of the 13 PCGs, 3 genes began with ATA, 2 began with ATT 3 began with ATG and 3 began with GTG or TTG, while only *cox1* began with ACG. It is evident that 6 genes (*nad4l*, *cob*, *cox1*, *nad2*, *atp6* and *atp8*) share a stop codon TAA. In addition, 3 genes (*nad6*, *cox2* and *cox3*) have a TAG stop codon, while 4 genes (*nad1*, *nad3, nad5* and *nad4*) have incomplete stop codons T (Table 3). The putative control region (322 bp) is located between *nad1* and *cob*.

**Figure 4:**
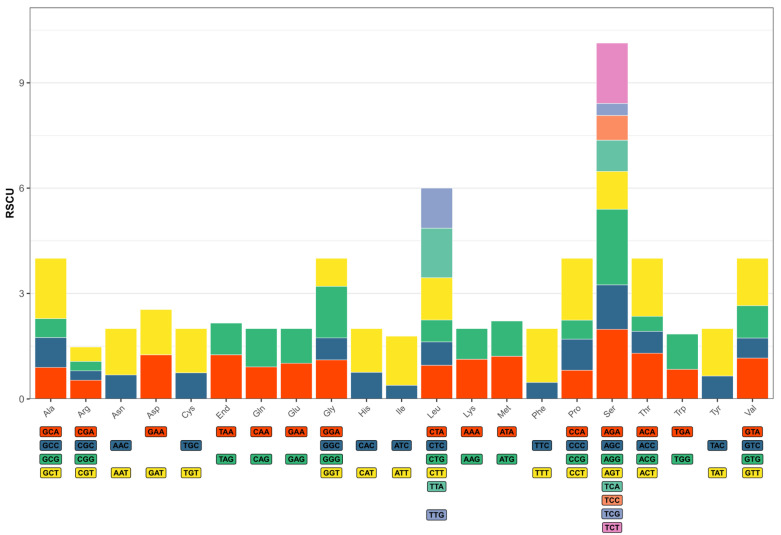
Relative synonymous codon usage (RSCU) of the mitochondrial genome of *Portunion sinensis*. The bottom graphic shows all the sense codons used for each amino acid, with the height of each column representing the sum of the RSCU values of all the codons.

The RSCU and codon numbers of PCGs are listed in Table S1 and illustrated in Figure 4. Of these codons, TTT is the most frequently used, appearing 237 times in total, while CGC is the least frequently used, appearing only 17 times. Of the 31 codons in mitochondrial DNA with RSCU values exceeding 1, 10 end with A, 14 end with T, one ends with C and six end with G. This indicates a preference for codons ending with A and T.

**Figure 5:**
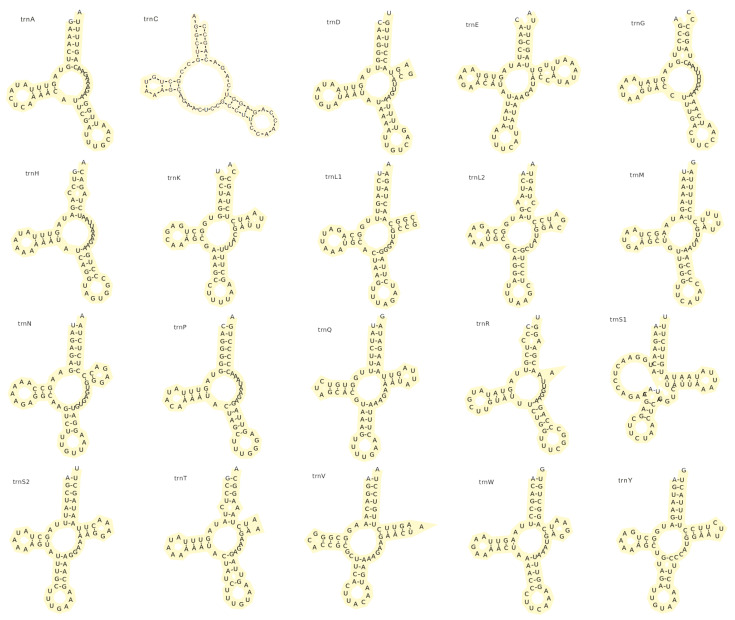
Secondary structures of the 20 mitochondrial tRNAs encoded by *Portunion sinensis*.

The total length of rRNA in the *P. sinensis* mitochondrial DNA sample is 1841 base pairs, with AT and GC skew values of 0.071 and −0.027, respectively. The mitochondrial genome contains 20 tRNAs in total, with the absence of *trnF* and *trnI*, resulting in a total length of 1189 base pairs. The AT skew value for these tRNAs is 0.060, while the GC skew value is notably high at 0.130. Figure 5 shows the secondary structures of the tRNAs encoded by the mitochondrion. Notably, *trnA*, *trnC*, *trnG*, *trnH*, *trnP* and *trnR* lack the T-arm structure and *trnS1* lacks the D-arm structure; all exhibit atypical cloverleaf secondary structural features.

### 3.3. Phylogenetic Relationships

**Figure 6:**
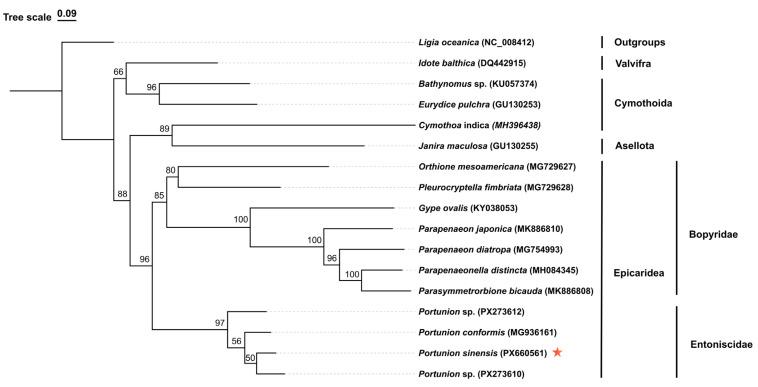
Phylogenetic tree of *Portunion sinensis* and related species constructed from the concatenated sequences of 13 PCGs and 2rRNAs (by maximum likelihood, ML). The ML bootstrap support values are denoted at each node. The asterisk (*) indicates the species sequenced in this study.

The phylogenetic reconstruction of the family Bopyridae using ML methods was based on the concatenated dataset of 13 protein-coding genes and 2 ribosomal RNAs (PCGs + rRNAs) (Figure 6). The analyses recovered the genus *Portunion* as a coherent lineage within Entoniscidae. Entoniscidae and Bopyridae together formed a strongly supported monophyletic group corresponding to the suborder Epicaridea. In contrast, resolution within the genus *Portunion* was limited. The internal nodes connecting the sampled species received low bootstrap support (BS < 60). This lack of resolution is likely attributable to the limited informative sites provided by the partial sequences included in the alignment (Table 1). Despite the low support for interspecific relationships, *P. sinensis* formed a distinct lineage genetically differentiated from other congeners.

The genetic distances calculated based on the Kimura 2-parameter (K2P) model are presented [31]. The average genetic distances among *P. sinensis* individuals collected from the coastal waters of China ranged from 0.0% to 0.1%. In contrast, the average genetic distance between *P. sinensis* and the known congeneric species *P. conformis* was 14.0%, while the distances to the Vietnam samples (PX273610 and PX273612) were 11.7% and 18.2%, respectively. Consequently, *P. sinensis* meets the “standard sequence threshold” proposed by Hebert et al. [14], supporting its recognition as a distinct species at the molecular level and corroborating its taxonomic status established based on morphology [32].

## 4. Discussion

### 4.1. Phylogenetic Relationships and Molecular Validation of Species Status

Based on the phylogenetic tree, the family Entoniscidae formed a well-supported monophyletic clade, which corresponds with the classification results based on parasitic habits that separate the unique endoparasitic Entoniscidae from other ectoparasitic groups [33]. As to whether Entoniscidae is an early independently diverged branch or a monophyletic branch sister to Bopyridae, support from molecular sequences of additional families is required.

Regarding the genus *Portunion*, the phylogenetic analysis revealed a polytomous structure with low bootstrap support for internal nodes. This lack of resolution is likely primarily attributable to the limited length of the available reference sequences used in the alignment. Most public data for *Portunion* species are restricted to partial *cox1* barcoding fragments, which provide insufficient phylogenetically informative sites to resolve deep interspecific branching orders. However, a contribution from rapid evolutionary radiation cannot be ruled out [34]. Theoretically, if speciation events occur in rapid succession, the synapomorphies accumulated by ancestral populations during short internodes may be insufficient to be captured by standard molecular markers, leading to poor phylogenetic resolution [35]. As current molecular data are insufficient to distinguish between methodological limitations and true biological radiation, future studies incorporating more genetic loci or complete genomes from closely related species are required.

### 4.2. Occurrence and Distribution of the Non-Canonical Start Codon in cox1

It is noteworthy that in the mitochondrial protein-coding genes of *P. sinensis*, the start codon of *cox1* is ACG rather than the standard ATG. The use of ACG does not exhibit phylogenetic continuity within Isopoda. It is found in some free-living suborders, such as Valvifera (e.g., *Idotea balthica*) and Oniscidea (e.g., *Ligia oceanica*) [36,37]; however, in the parasitic family Bopyridae, the closely related *Gyge ovalis* does not use this codon but retains the standard ATG [38], and ACG was observed as the *cox1* start codon only in *Pleurocryptella skinkai* of the same family [39].

This mosaic distribution pattern indicates that the non-canonical start codon ACG is not a stable synapomorphy of the parasitic clade [40], but is more likely a homoplastic trait resulting from independent parallel evolution or high mutational plasticity within Isopoda [41]. This implies that different groups more likely independently underwent T-to-C transition mutations rather than inheriting them from a common ancestor.

Although ACG is a non-canonical start codon, this does not imply a loss of translational function. Wolstenholme pointed out in a review of mitochondrial genomics that the use of various non-canonical start codons is a widespread phenomenon in metazoan mitochondria; this diversity is not a random error but a stable evolutionary strategy formed under the context of high A + T selection pressure, genome compaction, and tRNA co-evolution [42]. Regarding specific translation mechanisms, classic experimental studies by Peabody showed that ACG can efficiently initiate translation within a strong Kozak sequence context and exhibits the highest initiation efficiency among all single-base non-AUG variants [43]. Crucially, the polypeptide chain initiated at this site still starts with Methionine [44]. Therefore, future studies need to incorporate transcriptome sequencing (RNA-seq) alignment or protein N-terminal sequencing to definitively confirm the true start site of *cox1* and its translational initiation mechanism at the transcriptomic or proteomic level.

### 4.3. Structural Reduction and Putative Loss of Mitochondrial tRNAs

In the predicted mitochondrial tRNA secondary structures of this study, *trnA*, *trnC*, *trnG*, *trnH*, *trnP*, and *trnR* lack the typical TψC loop of the cloverleaf model, with the anticodon loop directly connected to the acceptor arm or variable loop; *trnS1* lacks the D-arm structure. All these tRNAs exhibit atypical cloverleaf secondary structural characteristics. Such structural simplification is not rare in animal mitochondrial genomes and has been frequently reported in invertebrates [45,46]. Although these truncated tRNAs deviate from the canonical secondary structure, this does not imply a loss of function. Research by Ohtsuki et al. indicates that such structural aberrations can be compensated by alternative mechanisms. Specifically, tRNAs lacking the D-arm can maintain the necessary spatial distance between the acceptor stem and the anticodon loop through unique intramolecular interactions, despite lacking the classical L-shape [47]. Furthermore, the functional loss of the T-arm may be compensated by specific post-transcriptional modifications (e.g., 1-methyladenosine) [48] or the co-evolution of mitochondrial elongation factors (EF-Tu) [49], which ensure efficient recognition and binding.

Compared with the isopod mitochondrial genome ground pattern [49], we found a putative loss of certain tRNA genes (*trnF* and *trnI*) in *P. sinensis*. This is consistent with the widespread tRNA gene loss observed by Kilpert across different isopod lineages. For instance, *trnI* is missing in the mitochondrial genome of *Gyge ovalis*, *trnF* is missing in *Eurydice pulchra* [50], and a more extensive loss of 9 tRNA genes occurs in the mitochondrial genome of *Armadillidium vulgare* [51]. Notably, this retention rate is significantly higher than that reported in the free-living *Armadillidium vulgare* (13 tRNAs) or the predatory *Eurydice pulchra* (16 tRNAs) [49]. This observation suggests that parasitic specialization is not linearly correlated with extensive gene loss in mitochondria [52].

On the contrary, the low gene counts reported in isopod lineages may partly stem from annotation artifacts rather than genuine biological loss. The high nucleotide substitution rates and structural aberrations in isopods may cause standard search algorithms (e.g., tRNAscan-SE) to fail in detecting tRNA genes [53]. Doublet systematically verified the expression of 13 tRNA genes in the *A. vulgare* mtDNA using RT-PCR and circularized RT-PCR (cRT-PCR) combined with cloning and sequencing [53]. This revealed that a truncated *trnH* undergoes extensive 3′-end repair and 5′-end addition of a G-1 residue post-transcriptionally. This also indicates that even under conditions of high mitochondrial genome compaction and extensive gene overlap [54], isopods can still maintain normal gene expression functions through complex RNA processing mechanisms, despite showing a prevalent loss of D-loops or T-loops, or even entire arms. Whether the *trnF* and *trnI* that were undetected by algorithm software in the *P. sinensis* mitochondrial genome also exist cryptically through similar mechanisms remains to be deeply analyzed in the future by combining transcriptome data.

## 5. Conclusions

Through morphological observation, the parasites collected and isolated in this study were identified as *Portunion sinensis*, a recently described species characterized by a unique body architecture where the anterior thorax is vertical and the female’s ventral processes are directed posteriorly. The complete mitochondrial genome was determined to be 14,603 bp in length, containing 35 genes: 13 protein-coding genes, 2 rRNA genes, 20 tRNA genes, and a putative control region (CR). The mitochondrial genome of *P. sinensis* exhibits a high degree of evolutionary streamlining and adaptation, characterized by several non-standard features. These include the use of an atypical ACG start codon for *cox1*, the presence of incomplete stop codons (T--) for several PCGs (e.g., *nad1*, *nad3, nad5 and nad4*), and significant structural reductions (such as the absence of the TψC-loop) in multiple tRNAs. Furthermore, *P. sinensis* shows putative tRNA gene losses (*trnF* and *trnI*), aligning with the broader pattern of tRNA gene attrition observed across diverse isopod lineages. Collectively, these genomic modifications underscore a pervasive evolutionary trend towards compactness and efficiency, likely driven by the intense selective pressures associated with a parasitic lifestyle. This study represents the first report on the mitochondrial genome of *Portunion sinensis*, filling a gap in the genomic data for the superfamily Bopyroidea. The results provide fundamental molecular data for the identification of parasites within the genus *Portunion* and offer valuable insights for the phylogenetic and taxonomic studies of the family Entoniscidae and the suborder Epicaridea.

## Figures and Tables

**Table 1:** Taxonomic information and GenBank entry numbers for all species used in the phylogenetic analysis.

Suborder	Family	Genus	Species	Accession Number
Epicaridea	Entoniscidae	*Portunion*	*Portunion* sp.	PX273610
		*Portunion*	*Portunion* sp.	PX273612
		*Portunion*	***Portunion sinensis***	**PX660561**
		*Portunion*	*Portunion co* *n* *formis*	MG936161
	Bopyridae	*Gy* *g* *e*	*Gy* *g* *e ovalis*	KY038053
		*Pleurpcrytella*	*Pleurocryptella fimbriata*	MG729628
		*Orthione*	*Orthione mesoamericana*	MG729627
		*Parapenaeon*	*Parapenaeon japoni* *c* *a*	MK886810
			*Parapenaeon diatropa*	MG754993
		*Parapenaeonella*	*Parapenaeonella distincta*	MH084345
		*Parasymmetrorbione*	*Parasymmetrorbione bicauda*	MK886808
Cymothoida	Cymothoidae	*Cymothoa*	*Cymothoa indica*	MH396438
	Cirolanidae	*Bathynomus*	*Bathynomus* sp.	KU057374
		*Eurydice*	*Eurydice pulchra*	GU130253
Valvifra	Idoteidae	*Idotea*	*Idotea balthica*	DQ442915
Asellota	Janirdae	*Janira*	*Janira maculosa*	GU130255
Oniscidea	Ligiidae	*Ligia*	*Ligia oceanica*	NC_008412

**Table 2:** Nucleotide composition and skewness of the whole mitochondrial genome and distinct gene categories (PCGs, tRNAs, and rRNAs) of *Portunion sinensis*.

*Portunion sinensis*	Size (bp)	A%	T%	G%	C%	A + T%	G + C%	AT-Skew	GC-Skew
Mitogenome	14,603	26.75	31.22	24.25	17.78	57.97	42.03	−0.077	0.154
PCGs	10,971	23.08	34.42	23.04	19.46	57.5	42.5	−0.197	0.084
tRNAs	1189	34.76	30.81	19.44	14.98	65.77	34.43	0.060	0.130
rRNAs	1841	31.5	27.32	20.04	21.13	58.82	41.17	0.071	−0.027

**Table 3:** Overview of the complete mitochondrial genome of *Portunion sinensis*.

Gene	Position		Length	Strand	Codon		Intergenic Length
	From	To			Start	Stop	
*cox1*	1	1539	1539	H	**ACG**	TAA	0
trnL2-TAA	1535	1593	59	H			−5
*cox2*	1594	2277	684	H	ATT	TAG	0
trnK-TTT	2280	2339	60	H			2
trnD-GTC	2338	2397	60	H			−2
*atp8*	2398	2556	159	H	TTG	TAA	0
*atp6*	2550	3221	672	H	GTG	TAA	−7
*cox3*	3221	4006	786	H	ATG	TAG	−1
trnG-TCC	4005	4062	58	H			−2
trnR-TCG	4061	4116	56	H			−2
*nad3*	4117	4465	349	H	ATT	T--	0
trnA-TGC	4466	4523	58	H			0
*nad1*	4537	5476	940	L	ATG	T--	13
trnL1-TAG	5459	5518	60	L			−18
trnN-GTT	5515	5575	61	H			−4
rrnS	5575	6273	699	H			−1
trnW-TCA	6287	6346	60	L			13
trnS1-TCT	6405	6468	64	L			58
trnE-TTC	6468	6534	67	L			−1
D-loop	6535	6856	322				0
*cob*	6860	7990	1131	L	ATA	TAA	220
trnT-TGT	8066	8123	58	L			75
*nad5*	8125	9847	1723	H	ATA	T--	1
trnH-GTG	9910	9967	58	L			62
*nad4*	9968	11,300	1333	L	ATG	T--	0
*nad4l*	11,301	11,582	282	L	ATA	TAA	0
trnP-TGG	11,583	11,638	56	L			0
*nad6*	11,640	12,122	483	H	GTG	TAG	1
trnS2-TGA	12,121	12,180	60	H			−2
rrnL	12,192	13,332	1141	L			11
trnV-TAC	13,318	13,378	61	L			−15
trnQ-TTG	13,377	13,436	60	L			−2
trnM-CAT	13,463	13,522	60	H			26
*nad2*	13,574	14,506	933	H	TTG	TAA	24
trnC-TGC	14,492	14,543	52	L			−15
trnY-GTA	14,543	14,603	61	L			−1

## Data Availability

All figures and tables used to support the results of this study have been included. The data presented in this study are openly available in GenBank at [https://www.ncbi.nlm.nih.gov/ (accessed on 25 January 2026)], reference number [PX660561].

## Supplementary Materials

The following supporting information can be downloaded at: https://www.mdpi.com/article/10.3390/biology15030282/s1, Table S1: Relative synonymous codon usage and codon number of *Portunion sinensis* mitochondrial PCGs.
