# Supplementary material for: The Complete Mitochondrial Genome of Portunion sinensis (Crustacea: Isopoda) and Its Phylogenies
Source: Biology (Basel). 2026 Feb 4;15(3):282. doi: 10.3390/biology15030282 (PMC12896808; doi:10.3390/biology15030282)
Supplement: Supplementary file 1 [file biology-15-00282-s001.zip › biology-4098735-supplementary.pdf]

Table S1. Relative synonymous codon usage and codon number of *Portunion sinensis* mitochondrial PCGs.

AmAcid	Codon	Count	RSCU
End	UAG	65	0.899
End	UAA	91	1.258
Ala	GCG	40	0.537
Ala	GCA	67	0.899
Ala	GCU	128	1.718
Ala	GCC	63	0.846
Cys	UGU	79	1.254
Cys	UGC	47	0.746
Asp	GAU	63	1.286
Asp	GAA	36	1.258
Glu	GAG	90	0.984
Glu	GAA	93	1.016
Phe	UUU	237	1.529
Phe	UUC	73	0.471
Gly	GGG	135	1.471
Gly	GGA	102	1.112
Gly	GGU	73	0.796
Gly	GGC	57	0.621
His	CAU	44	1.239
His	CAC	27	0.761
Ile	AUU	125	1.394
Ile	AUC	35	0.39
Lys	AAG	103	0.869
Lys	AAA	134	1.131
Leu	UUG	126	1.14
Leu	UUA	156	1.412
Leu	CUG	69	0.624
Leu	CUA	106	0.959
Leu	CUU	133	1.204
Leu	CUC	73	0.661
Met	AUG	65	1
Met	AUA	109	1.216
Asn	AAU	102	1.316
Asn	AAC	53	0.684
Pro	CCG	27	0.54
Pro	CCA	41	0.82
Pro	CCU	88	1.76
Pro	CCC	44	0.88
Gln	CAG	76	1.086
Gln	CAA	64	0.914
Arg	CGG	42	0.267
Arg	CGA	34	0.534
Arg	CGU	26	0.408
Arg	CGC	17	0.267
Ser	AGG	137	2.152
Ser	AGA	126	1.979
Ser	AGU	69	1.078
Ser	AGC	81	1.266
Ser	UCG	22	0.344
Ser	UCA	57	0.891
Ser	UCU	110	1.719

Ser	UCC	45	0.703
Thr	ACG	19	0.425
Thr	ACA	58	1.296
Thr	ACU	74	1.654
Thr	ACC	28	0.626
Val	GUG	65	0.922
Val	GUA	82	1.163
Val	GUU	95	1.348
Val	GUC	40	0.567
Trp	UGG	73	1
Trp	UGA	61	0.843
Tyr	UAU	113	1.345
Tyr	UAC	55	0.655
